# Supplementary material for: Content-rich biological network constructed by mining PubMed abstracts
Source: BMC Bioinformatics. 2004 Oct 8;5:147. doi: 10.1186/1471-2105-5-147 (PMC528731; doi:10.1186/1471-2105-5-147)
Supplement: Additional File 5 — The original Chilibot query results of the term "long-term potentiation (LTP)" and 22 other terms, limiting the latest references analyzed to the years 1990, 1995, 2000, and 2004. [file 1471-2105-5-147-S5.bz2 › chilibotAdditionalFile5/ltp1995/html/PKC_ACTIN.html]

 


 **PKC** and **ACTIN** 
  
Found 137 abstracts in PubMed,  **30 abstracts were retrieved and analyzed**.  


---

 Search Google  |
 PDF files only 
|  EDU domain only 

---

**Interactive relationship** (e.g. stimulation, inhibition, etc)

- We found that cells injected with active  **PKC**  alpha expressed increased amounts of  **actin**  compared with control cells.  Ref: 8001276 Circ Res, 1995
- Dephosphorylation by calcineurin restored  **actin**  binding and  **actin**  activated myosin MgATPase inhibition which had been reduced by  **PKC**  catalyzed phosphorylation.  Ref: 7619814 Biochemistry, 1995
- Myristoylated, alanine rich C kinase substrate MARCKS is a lipopolysaccharide induced protein kinase C  **PKC**  substrate that has been proposed to regulate  **actin**  membrane interactions, as well as  **actin**  structure at the membrane.  Ref: 7650489 J Exp Med, 1995
- Cross linking of surface IgM or direct activation of  **PKC**  with PMA resulted in time and concentration dependent increases in F  **actin**  content, MAPK p42erk 2 activation, and phosphorylation of p90rsk.  Ref: 7733965 Biochem Biophys Res Commun, 1995
- Although the kinetics are different depending on the stimulant, activation of  **PKC**  using multivalent antigen, PMA or diC8 also causes the polymerization of  **actin**  and an increase in the F  **actin**  content of the cells.  Ref: 7749199 Mol Biol Cell, 1995
- A tonic contraction is initiated when the DAG generated during PIP2 hydrolysis binds to and activates protein kinase C  **PKC**  in the presence of increased cytosolic calcium, leading to shortening of the  **actin**  filaments.  Ref: 7673079 J Anim Sci, 1995
- Inhibitors of  **PKC**  reduced zymosan binding to the macrophage surface and blocked the focal accumulation of F  **actin** , talin, phosphotyrosine containing proteins, MARCKS, and  **PKC**  alpha beneath attached particles.  Ref: 7650489 J Exp Med, 1995
- Prolonged treatment of RD cells with the protein kinase C  **PKC**  activator 12 O tetradecanoylphorbol 13 acetate TPA induces growth arrest and myogenic differentiation as shown by the accumulation of alpha  **actin**  and myosin light and heavy chains, without affecting the expression of MyoD and myogenin.  Ref: 7547506 Cell Growth Differ, 1995
- Treatment of mouse eggs from either CF 1 or CD 1 mice with these activators of  **PKC**  did not result in the formation of these polar body like structures, did not cause an increase in filamentous  **actin** , and did not result in a reduction in histone H1 kinase activity.  Ref: 7649380 Dev Biol, 1995
- The  **PKC**  inhibitor Ro 31 8220 elicits a high proportion of polarized ED50 = 1.5 x 10 6 M and locomoting cells and reduces the relative amount of F  **actin**  by 29% at 10 5 M in initially resting cells.  Ref: 7536787 J Leukoc Biol, 1995
- This indicates that the spherical shape and the F  **actin**  content of resting cells are maintained by constitutive  **PKC**  activity.  Ref: 7536787 J Leukoc Biol, 1995
- MARCKS,  **PKC**  alpha, and myosin I colocalized with F  **actin**  and talin in the cortical cytoplasm adjacent to forming phagocytic cups.  Ref: 7650489 J Exp Med, 1995
- The results suggest that protein kinase C  **PKC**  plays a pivotal role in the control of F  **actin**  levels, locomotion, pinocytosis, and cell shape in lymphocytes.  Ref: 7536787 J Leukoc Biol, 1995
- An identical cytochalasin B sensitive  **actin**  driven response is caused by treating of the cells with the protein kinase C  **PKC**  activator 12 myristate 13 acetate.  Ref: 7656899 Eur J Cell Biol, 1995
- The specific nature of the slow component is not certain but may involve low level  **PKC**  activity and require the integrity and capability for remodeling of a specific portion of the  **actin**  containing cytoskeleton.  Ref: 7943389 Am J Physiol, 1994
- In contrast, phosphorylation by CK II or  **PKC**  inhibited the binding to  **actin** .  Ref: 7818551 Biochem Biophys Res Commun, 1995
- Furthermore, depletion of  **PKC**  activity through long term exposure to PMA, inhibited both the F  **actin**  response and the increased synthesis of PIP and PIP2 induced by either DNP BSA or diC8.  Ref: 7749199 Mol Biol Cell, 1995

**Parallel relationship** (e.g. studied together, co-existance, homology, etc.)

- Furthermore, we show that zeta  **PKC**  has a role in maintaining the integrity of the  **actin**  cytoskeletal structure in IL 2 stimulated cells.  Ref: 7589144 Eur J Immunol, 1995
- A role for both protein kinase C and cytoskeletal rearrangements in CD2 regulation of integrin activity is also suggested, since a  **PKC**  inhibitor partially inhibits CD2 induced increases in beta 1 integrin function, and CD2 stimulation increases F  **actin**  content in a wortmannin sensitive manner.  Ref: 8557753 J Cell Biol, 1995
- On the contrary, zeta  **PKC**  is not involved in the  **actin**  cytoskeleton organization when cells are maintained in IL 4, confirming our previous results showing that IL 4 induced signal transduction is  **PKC**  independent.  Ref: 7589144 Eur J Immunol, 1995
- MARCKS binds calcium calmodulin and crosslinks F  **actin** , and both these activities are regulated by  **PKC**  dependent phosphorylation.  Ref: 7720702 EMBO J, 1995
- In addition,  **PKC**  alpha accumulates in the endoplasmic reticulum,  **PKC**  beta II associates with  **actin**  rich microfilaments of the cytoskeleton,  **PKC**  gamma accumulates in Golgi organelles, and  **PKC**  epsilon associates with nuclear membranes.  Ref: 7730383 J Biol Chem, 1995
- Incubating the cells with the protein kinase C inhibitor bisindolylmaleide or staurosporine inhibits both the muscarinic receptor mediated and phorbolester mediated  **actin**  driven response, suggesting that activated  **PKC**  plays a crucial role.  Ref: 7656899 Eur J Cell Biol, 1995
- Calponin is phosphorylated by protein kinase C  **PKC**  and calcium calmodulin dependent protein kinase II CaM kinase II, primarily at S175, with loss of  **actin**  binding and inhibition of the  **actin**  activated myosin MgATPase.  Ref: 7619814 Biochemistry, 1995
- We report here, using confocal microscopy, that IL 2 induces expression, translocation and association of zeta  **PKC**  to a structure coincident with the  **actin**  cytoskeleton.  Ref: 7589144 Eur J Immunol, 1995
- These results and the recent finding that p50 binds to  **actin**  fibers raise the possibility that p50 controls the binding of  **actin**  fibers to the plasma membrane under regulation by  **PKC**  in T cells.  Ref: 7775393 J Biochem (Tokyo), 1995
- Selective responses  **actin**  polymerization, shape changes, locomotion, pinocytosis to the  **PKC**  inhibitor Ro 31 8220 suggest that  **PKC**  discriminately regulates functions of human blood lymphocytes.  Ref: 7536787 J Leukoc Biol, 1995
